# The genome sequence of the false flower beetle, *Anaspis regimbarti *Schilsky, 1895

**DOI:** 10.12688/wellcomeopenres.23737.1

**Published:** 2025-02-19

**Authors:** Maxwell V. L. Barclay, Dmitry Telnov, Natural History Museum Genome Acquisition Lab, Darwin Tree of Life Barcoding collective, Wellcome Sanger Institute Tree of Life Management, Samples and Laboratory team, Wellcome Sanger Institute Scientific Operations: Sequencing Operations, Wellcome Sanger Institute Tree of Life Core Informatics team, Tree of Life Core Informatics collective, Darwin Tree of Life Consortium

**Affiliations:** 1 Natural History Museum, London, England, UK; 2 Daugavpils University, Daugavpils, Latvia; 3 Institute of Biology, University of Latvia, Rīga, Latvia

**Keywords:** Anaspis regimbarti, false flower beetle, genome sequence, chromosomal, Coleoptera

## Abstract

We present a genome assembly from a specimen of *Anaspis regimbarti* (the false flower beetle; Arthropoda; Insecta; Coleoptera; Scraptiidae). The genome sequence has a total length of 457.61 megabases. Most of the assembly (99.89%) is scaffolded into 8 chromosomal pseudomolecules, including the X sex chromosome. The mitochondrial genome has also been assembled and is 16.39 kilobases in length.

## Species taxonomy

Eukaryota; Opisthokonta; Metazoa; Eumetazoa; Bilateria; Protostomia; Ecdysozoa; Panarthropoda; Arthropoda; Mandibulata; Pancrustacea; Hexapoda; Insecta; Dicondylia; Pterygota; Neoptera; Endopterygota; Coleoptera; Polyphaga; Cucujiformia; Tenebrionoidea; Scraptiidae; *Anaspis*; *Anaspis regimbarti* Schilsky, 1895 (NCBI:txid346699)

## Background

Scraptiidae (false flower beetles) is a small family of Tenebrionoidea. Two subfamilies, Scraptiinae and Anaspidinae, are recognised in Scraptiidae, both of cosmopolitan distribution ( Lawrence & Ślipiński, 2010). While adult representatives of the subfamily Scraptiinae usually dwell in tree foliage and in cracks of tree bark, adults of the subfamily Anaspidinae are usually found on flowering shrubs and other plants ( Duff, 2020; Levey, 2009, and references therein). The English name of the family, false flower beetles, refers to the ecology of Scraptiinae rather than that of Anaspidinae and likely also highlights the external similarity of the adult false flower beetles with other common flower-visiting coleopterans such as tumbling flower beetles (Mordellidae). The Palaearctic species of the genus *Anaspis* are placed in six subgenera ( Iwan & Kubisz, 2020). There are about 127 Anaspis species and subspecies in the Palaearctic Region (not counting one dubious species) of which 79 occur in geographical Europe ( Iwan & Kubisz, 2020) and 11 species in two subgenera represent the British fauna ( Duff, 2018).

*Anaspis regimbarti* Schilsky, 1895 is placed in the false flower beetle subfamily Anaspidinae tribe Anaspidini ( Iwan & Kubisz, 2020). The species strongly resembles its congeners in external morphology and the fusiform body shape but is different in the shape and structure of the male genital organs and terminalia, the presence of darker setae along the elytral suture contrasting to pale setation on most of the elytral disc, the nearly equally wide antennomeres 3–5 and the pale rufous forehead and pronotum and the yellowish to pale rufous pro- and mesothoracic legs ( Duff, 2018; Levey, 2009). The pronotum can be darkened in some specimens, which were described by Joy (1912), based on specimens from Bradfield, Berkshire, England as “variety *fraudulenta*”, but this form is not presently treated as taxonomically distinct.

The morphological features of *Anaspis* species often vary, especially the body colouration, the larvae are unknown for most the species and identification of taxa, especially those from outside central and northern Europe, is often challenging. It is hoped that genomic information may resolve some taxonomical uncertainties among *Anaspis* that have not yet been fully resolved by the combination of morphological and standard molecular techniques.

*Anaspis regimbarti* is a western Palaearctic species widely distributed in Western and Central Europe eastwards to eastern parts of Germany and northwards to southern Sweden; in the Mediterranean there are few records from southern France (including Corsica), Italy (including Sicily), Spain ( Franciscolo, 1995; GBIF Secretariat, 2024; Iwan & Kubisz, 2020) and the range extends towards Algeria in northern Africa ( Levey, 2009). Iwan and Kubisz (2020) list the species from 12 European countries, Belgium, Denmark, France, Germany, Ireland, Italy, the Netherlands, Portugal, Spain, Sweden, Switzerland, and the United Kingdom but omit or intentionally ignore the Algerian record (see above).

Larvae of *A. regimbarti* are saproxylic and develop in decaying wood of various deciduous and coniferous trees and are likely polyphagous, adults reared from decaying wood of *Betula* spp., *Fagus* sp., *Salix* sp. ( Koch, 1989), *Larix* spp. and *Quercus* spp. ( Levey, 2009, and references therein). The species is described as stenotopic, arboricol and floricol ( Koch, 1989). Adult beetles are anthophilous and occur on blossom of various trees and bushes, in Britain especially on *Crataegus* species ( Duff, 2020). In Britain, adults have been reported from April to August ( Duff, 2020) and the sequenced specimen was sampled at the beginning of May.

*Anaspis regimbarti* is a common and widespread species in United Kingdom and recorded in England, Wales and Scotland North to West Ross as well as in Ireland ( Duff, 2020; Levey, 2009). The species was not listed in the national Red Data Book ( Shirt, 1987), the present status of the species in the United Kingdom is Least Concern (LC) ( Alexander *et al.*, 2014).

**Figure 1.**
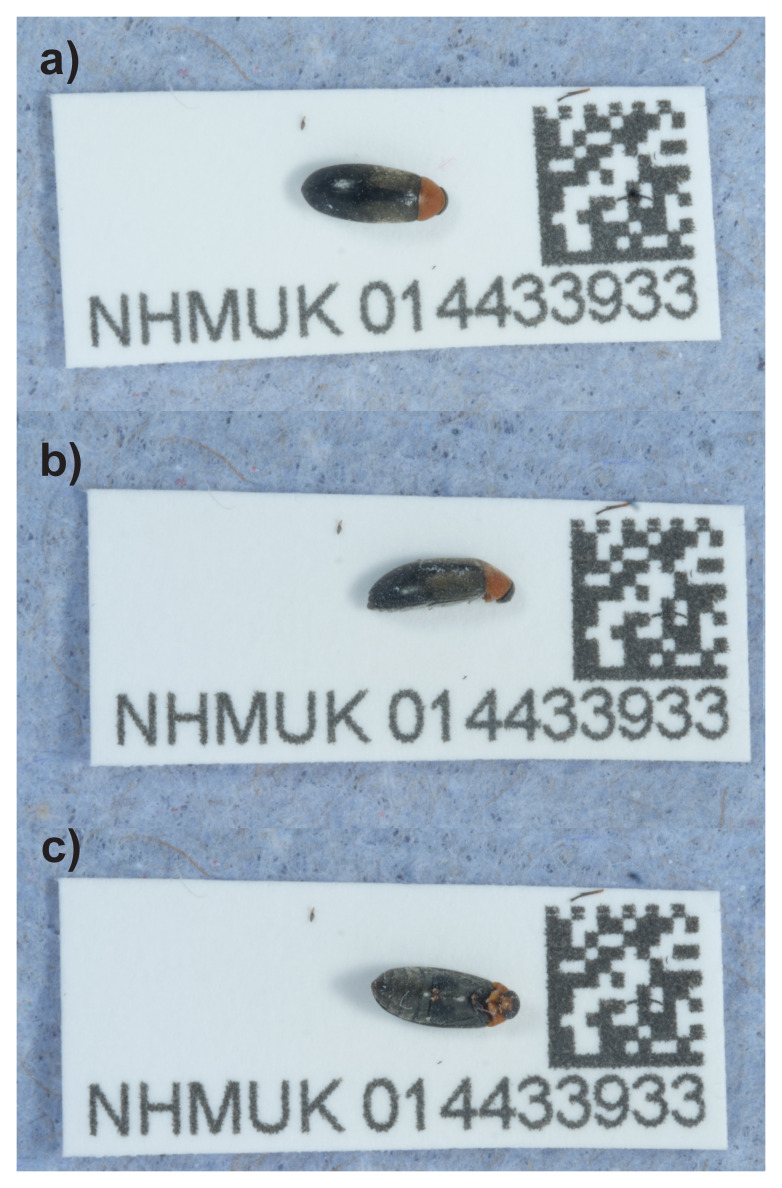
Photograph of the *Anaspis regimbarti* (icAnaRegi1) specimen used for genome sequencing.

Here we present a chromosomal-level genome sequence for *Anaspis regimbarti*, based on a female specimen from Tynemouth Street, London, England, United Kingdom ( Figure 1). The specimen used for sequencing was a typically-coloured female with rufous pronotum, collected from street trees in Tynemouth Street, Fulham, London, England by M.V.L. Barclay on 04.v.2021

## Genome sequence report

### Sequencing data

The genome of a specimen of *Anaspis regimbarti* ( Figure 1) was sequenced using Pacific Biosciences single-molecule HiFi long reads, generating 22.74 Gb from 2.27 million reads. GenomeScope analysis of the PacBio HiFi data estimated the haploid genome size at 449.96 Mb, with a heterozygosity of 0.86% and repeat content of 38.43%. These values provide an initial assessment of genome complexity and the challenges anticipated during assembly. Based on this estimated genome size, the sequencing data provided approximately 48.0x coverage of the genome. Chromosome conformation Hi-C data produced 140.75 Gb from 932.11 million reads. Table 1 summarises the specimen and sequencing information, including the BioProject, study name, BioSample numbers, and sequencing data for each technology.

**Table 1.** Specimen and sequencing data for *Anaspis regimbarti*.

Project information			
**Study title**	Anaspis regimbarti		
**Umbrella BioProject**	PRJEB71244		
**Species**	*Anaspis regimbarti*		
**BioSample**	SAMEA111458298		
**NCBI taxonomy ID**	346699		
Specimen information			
**Technology**	**ToLID**	**BioSample ** **accession**	**Organism part**
**PacBio long read sequencing**	icAnaRegi1	SAMEA111458347	Thorax and abdomen
**Hi-C sequencing**	icAnaRegi2	SAMEA114806140	whole organism
Sequencing information			
**Platform**	**Run accession**	**Read count**	**Base count (Gb)**
**Hi-C Illumina NovaSeq X**	ERR13317812	9.32e+08	140.75
**PacBio Sequel IIe**	ERR12370397	2.27e+06	22.74

### Assembly statistics

The primary haplotype was assembled, and contigs corresponding to an alternate haplotype were also deposited in INSDC databases. The assembly was improved by manual curation, which corrected 42 misjoins or missing joins. These interventions decreased the scaffold count by 16.0%. The final assembly has a total length of 457.61 Mb in 20 scaffolds, with 104 gaps, and a scaffold N50 of 64.26 Mb ( Table 2).

**Table 2.** Genome assembly data for *Anaspis regimbarti*.

Genome assembly		
Assembly name	icAnaRegi1.1	
Assembly accession	GCA_964204715.1	
*Alternate haplotype * *accession*	*GCA_964204705.1*	
Assembly level for primary assembly	chromosome	
Span (Mb)	457.61	
Number of contigs	124	
Number of scaffolds	20	
Longest scaffold (Mb)	96.37	
Assembly metrics	Measure	*Benchmark*
Contig N50 length	6.91 Mb	*≥ 1 Mb*
Scaffold N50 length	64.26 Mb	*= chromosome N50*
Consensus quality (QV)	Primary: 66.4; alternate: 66.4; combined 66.4	*≥ 40*
*k*-mer completeness	Primary: 80.68%; alternate: 80.19%; combined: 99.18%	*≥ 95%*
BUSCO *	C:98.4%[S:97.6%,D:0.9%], F:0.3%,M:1.2%,n:2,124	*S > 90%*, *D < 5%*
Percentage of assembly mapped to chromosomes	99.9%	*≥ 90%*
Sex chromosomes	X	*localised * *homologous pairs*
Organelles	Mitochondrial genome: 16.39 kb	*complete single * *alleles*

* BUSCO scores based on the endopterygota_odb10 BUSCO set using version 5.5.0. C = complete [S = single copy, D = duplicated], F = fragmented, M = missing, n = number of orthologues in comparison.

**Figure 2.**
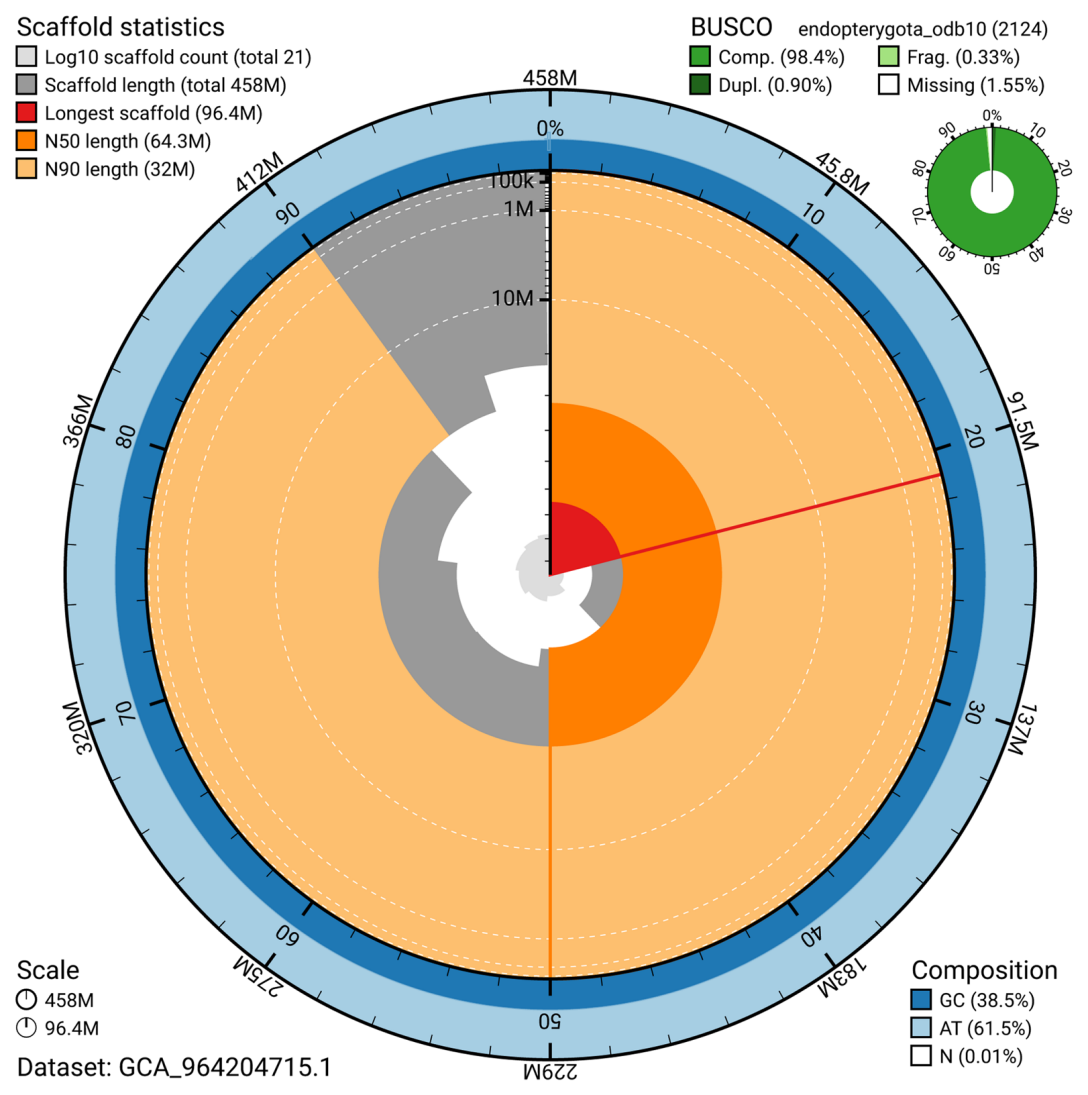
Genome assembly of *Anaspis regimbarti* icAnaRegi1.1: metrics. The BlobToolKit snail plot provides an overview of assembly metrics and BUSCO gene completeness. The circumference represents the length of the whole genome sequence, and the main plot is divided into 1,000 bins around the circumference. The outermost blue tracks display the distribution of GC, AT, and N percentages across the bins. Scaffolds are arranged clockwise from longest to shortest and are depicted in dark grey. The longest scaffold is indicated by the red arc, and the deeper orange and pale orange arcs represent the N50 and N90 lengths. A light grey spiral at the centre shows the cumulative scaffold count on a logarithmic scale. A summary of complete, fragmented, duplicated, and missing BUSCO genes in the endopterygota_odb10 set is presented at the top right. An interactive version of this figure is available at https://blobtoolkit.genomehubs.org/view/GCA_964204715.1/dataset/GCA_964204715.1/snail.

**Figure 3.**
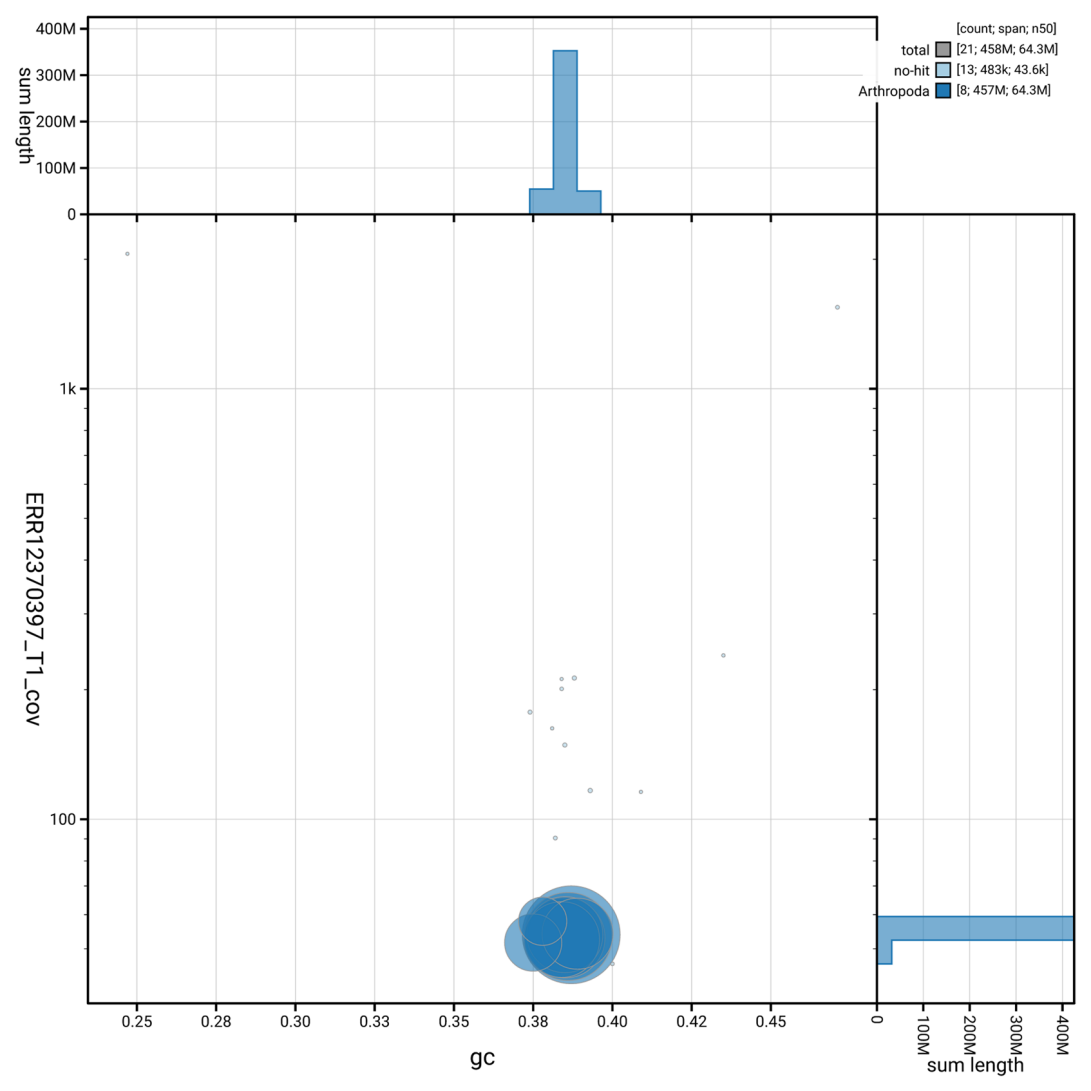
Genome assembly of *Anaspis regimbarti* icAnaRegi1.1: BlobToolKit GC-coverage plot. Blob plot showing sequence coverage (vertical axis) and GC content (horizontal axis). The circles represent scaffolds, with the size proportional to scaffold length and the colour representing phylum membership. The histograms along the axes display the total length of sequences distributed across different levels of coverage and GC content. An interactive version of this figure is available at https://blobtoolkit.genomehubs.org/view/GCA_964204715.1/blob.

**Figure 4.**
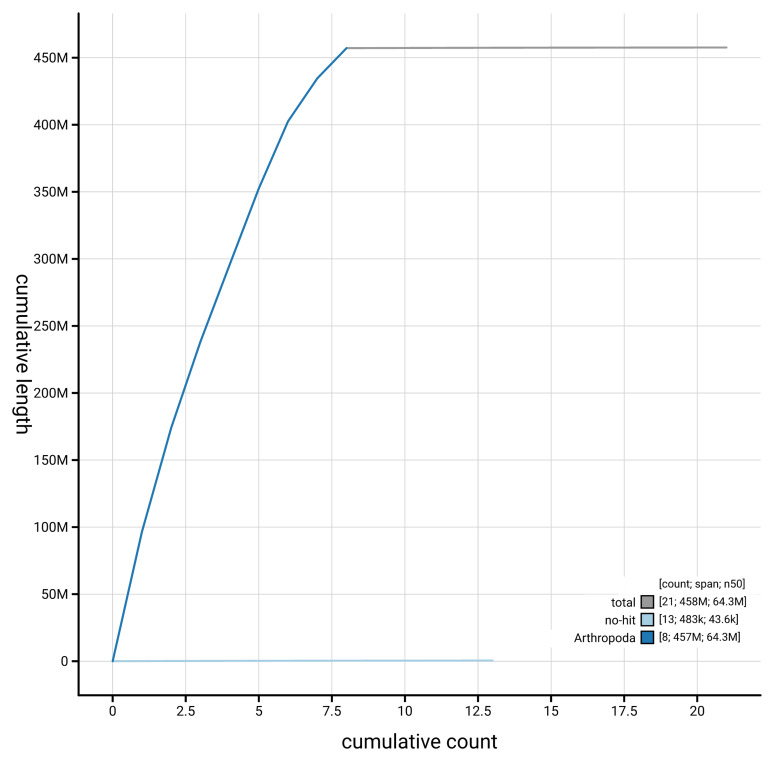
Genome assembly of *Anaspis regimbarti* icAnaRegi1.1: BlobToolKit cumulative sequence plot. The grey line shows cumulative length for all scaffolds. Coloured lines show cumulative lengths of scaffolds assigned to each phylum using the buscogenes taxrule. An interactive version of this figure is available at https://blobtoolkit.genomehubs.org/view/GCA_964204715.1/dataset/GCA_964204715.1/cumulative.

The snail plot in Figure 2 provides a summary of the assembly statistics, indicating the distribution of scaffold lengths and other assembly metrics. Figure 3 shows the distribution of scaffolds by GC proportion and coverage. Figure 4 presents a cumulative assembly plot, with separate curves representing different scaffold subsets assigned to various phyla, illustrating the completeness of the assembly.

**Figure 5.**
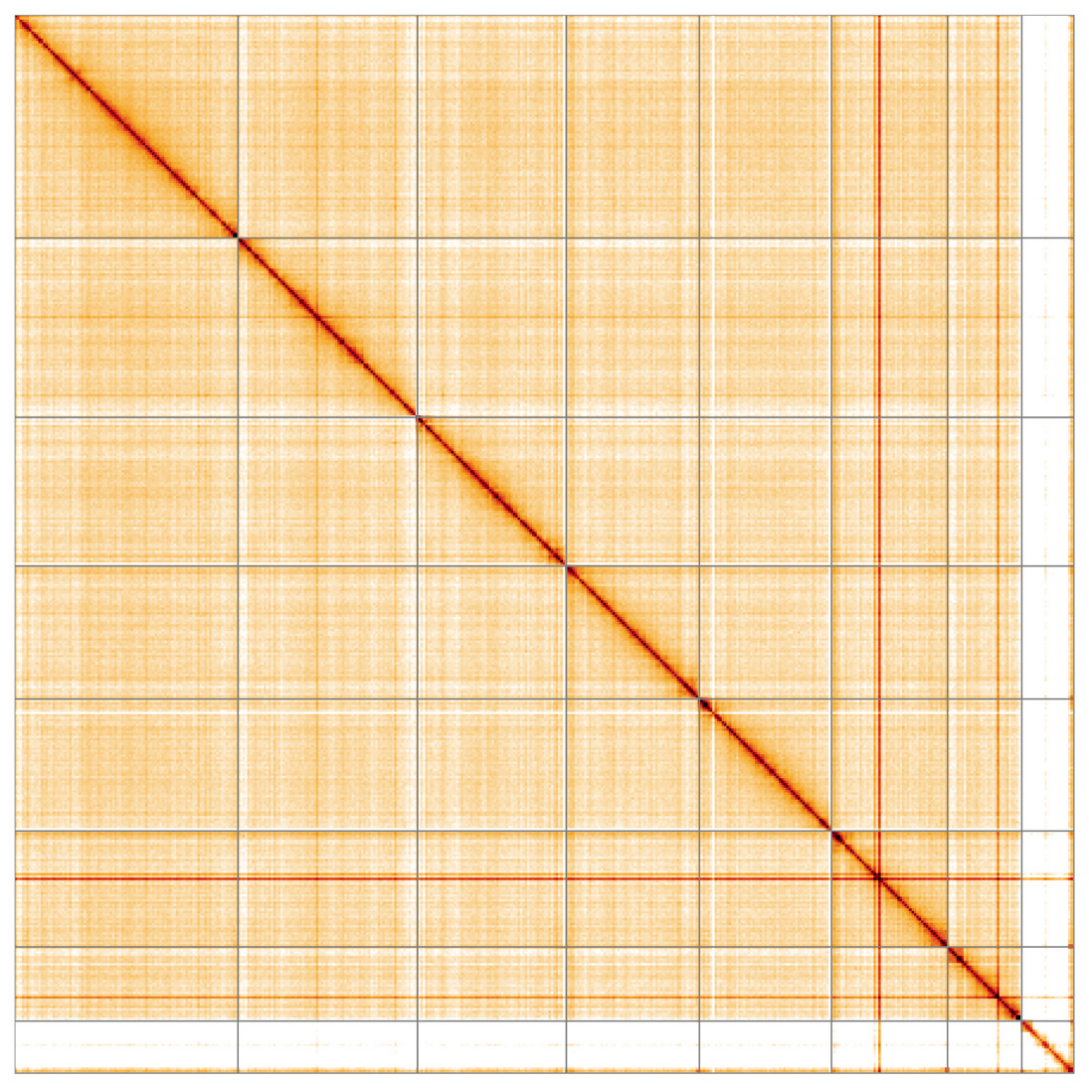
Genome assembly of *Anaspis regimbarti*: Hi-C contact map of the icAnaRegi1.1 assembly, visualised using HiGlass. Chromosomes are shown in order of size from left to right and top to bottom. An interactive version of this figure may be viewed at https://genome-note-higlass.tol.sanger.ac.uk/l/?d=MMpgYDACRXy2zujvaR_RdQ.

Most of the assembly sequence (99.9%) was assigned to 8 chromosomal-level scaffolds, representing 7 autosomes and the X sex chromosome. These chromosome-level scaffolds, confirmed by Hi-C data, are named according to size ( Figure 5; Table 3). During curation, chromosome X was assigned by synteny to the genome of *Anaspis maculata* (GCA_949128115.1) ( Telfer *et al.*, 2024).

**Table 3.** Chromosomal pseudomolecules in the genome assembly of *Anaspis regimbarti*, icAnaRegi1.

INSDC accession	Name	Length (Mb)	GC%
OZ123342.1	1	96.37	38.5
OZ123343.1	2	77.48	38.5
OZ123344.1	3	64.26	38.5
OZ123345.1	4	57.42	38.5
OZ123346.1	5	57.03	38.5
OZ123347.1	6	50.02	39
OZ123348.1	7	32.02	37.5
OZ123349.1	X	22.54	38
OZ123350.1	MT	0.02	25

The mitochondrial genome was also assembled. This sequence is included as a contig in the multifasta file of the genome submission and as a standalone record in GenBank.

### Assembly quality metrics

The estimated Quality Value (QV) and *k*-mer completeness metrics, along with BUSCO completeness scores, were calculated for each haplotype and the combined assembly. The QV reflects the base-level accuracy of the assembly, while *k*-mer completeness indicates the proportion of expected *k*-mers identified in the assembly. BUSCO scores provide a measure of completeness based on benchmarking universal single-copy orthologues.

The primary haplotype has a QV of 66.4, and the combined primary and alternate assemblies achieve an estimated QV of 66.4. The *k*-mer completeness for the primary assembly is 80.68%, and for the alternate haplotype, 80.19%. The combined primary and alternate assemblies achieve a *k*-mer completeness of 99.18%. BUSCO analysis using the endopterygota_odb10 reference set ( *n* = 2,124) indicated a completeness score of 98.4% (single = 97.6%, duplicated = 0.9%).

Table 2 provides assembly metric benchmarks adapted from Rhie *et al.* (2021) and the Earth BioGenome Project Report on Assembly Standards September 2024. The primary assembly achieves the EBP reference standard of 6.C.Q66.

## Methods

### Sample acquisition and DNA barcoding

A female *Anaspis regimbarti* (specimen ID NHMUK014433933, ToLID icAnaRegi1) was collected from Tynemouth Street, London, England, United Kingdom (latitude 51.47, longitude –0.19) on 2021-05-04 by handpicking. The specimen used for Hi-C sequencing (specimen ID NHMUK014440619, ToLID icAnaRegi2) was collected from Wetherby Gardens, Kensington, London, England, United Kingdom (latitude 51.49, longitude -0.18) on 2022-05-25 by handpicking. Both specimens were collected and identified by Maxwell Barclay and preserved by dry freezing (–80 °C).

The initial identification was verified by an additional DNA barcoding process according to the framework developed by Twyford *et al.* (2024). A small sample was dissected from the specimen and stored in ethanol, while the remaining parts were shipped on dry ice to the Wellcome Sanger Institute (WSI). The tissue was lysed, the COI marker region was amplified by PCR, and amplicons were sequenced and compared to the BOLD database, confirming the species identification ( Crowley *et al.*, 2023). Following whole genome sequence generation, the relevant DNA barcode region was also used alongside the initial barcoding data for sample tracking at the WSI ( Twyford *et al.*, 2024). The standard operating procedures for Darwin Tree of Life barcoding have been deposited on protocols.io ( Beasley *et al.*, 2023).

Metadata collection for samples adhered to the Darwin Tree of Life project standards described by Lawniczak *et al.* (2022).

### Nucleic acid extraction

The workflow for high molecular weight (HMW) DNA extraction at the Wellcome Sanger Institute (WSI) Tree of Life Core Laboratory includes a sequence of procedures: sample preparation and homogenisation, DNA extraction, fragmentation and purification. Detailed protocols are available on protocols.io ( Denton *et al.*, 2023b). The icAnaRegi1 sample was prepared for DNA extraction by weighing and dissecting it on dry ice ( Jay *et al.*, 2023). Tissue from the thorax and abdomen was homogenised using a PowerMasher II tissue disruptor ( Denton *et al.*, 2023a). HMW DNA was extracted in the WSI Scientific Operations core using the Automated MagAttract v2 protocol ( Oatley *et al.*, 2023). The DNA was sheared into an average fragment size of 12–20 kb in a Megaruptor 3 system ( Bates *et al.*, 2023). Sheared DNA was purified by solid-phase reversible immobilisation, using AMPure PB beads to eliminate shorter fragments and concentrate the DNA ( Strickland *et al.*, 2023). The concentration of the sheared and purified DNA was assessed using a Nanodrop spectrophotometer and Qubit Fluorometer using the Qubit dsDNA High Sensitivity Assay kit. Fragment size distribution was evaluated by running the sample on the FemtoPulse system.

### Hi-C sample preparation

Tissue from the whole organism of the icAnaRegi2 sample was processed for Hi-C sequencing at the WSI Scientific Operations core, using the Arima-HiC v2 kit. In brief, 20–50 mg of frozen tissue (stored at –80 °C) was fixed, and the DNA crosslinked using a TC buffer with 22% formaldehyde concentration. After crosslinking, the tissue was homogenised using the Diagnocine Power Masher-II and BioMasher-II tubes and pestles. Following the Arima-HiC v2 kit manufacturer's instructions, crosslinked DNA was digested using a restriction enzyme master mix. The 5’-overhangs were filled in and labelled with biotinylated nucleotides and proximally ligated. An overnight incubation was carried out for enzymes to digest remaining proteins and for crosslinks to reverse. A clean up was performed with SPRIselect beads prior to library preparation. Additionally, the biotinylation percentage was estimated using the Qubit Fluorometer v4.0 (Thermo Fisher Scientific) and Qubit HS Assay Kit and Arima-HiC v2 QC beads.

### Library preparation and sequencing

Library preparation and sequencing were performed at the WSI Scientific Operations core.

***PacBio HiFi***

At a minimum, samples were required to have an average fragment size exceeding 8 kb and a total mass over 400 ng to proceed to the low input SMRTbell Prep Kit 3.0 protocol (Pacific Biosciences, California, USA), depending on genome size and sequencing depth required. Libraries were prepared using the SMRTbell Prep Kit 3.0 (Pacific Biosciences, California, USA) as per the manufacturer's instructions. The kit includes the reagents required for end repair/A-tailing, adapter ligation, post-ligation SMRTbell bead cleanup, and nuclease treatment. Following the manufacturer’s instructions, size selection and clean up was carried out using diluted AMPure PB beads (Pacific Biosciences, California, USA). DNA concentration was quantified using the Qubit Fluorometer v4.0 (Thermo Fisher Scientific) with Qubit 1X dsDNA HS assay kit and the final library fragment size analysis was carried out using the Agilent Femto Pulse Automated Pulsed Field CE Instrument (Agilent Technologies) and gDNA 55kb BAC analysis kit.

Samples were sequenced using the Sequel IIe system (Pacific Biosciences, California, USA). The concentration of the library loaded onto the Sequel IIe was in the range 40–135 pM. The SMRT link software, a PacBio web-based end-to-end workflow manager, was used to set-up and monitor the run, as well as perform primary and secondary analysis of the data upon completion.

***Hi-C***

For Hi-C library preparation, DNA was fragmented using the Covaris E220 sonicator (Covaris) and size selected using SPRISelect beads to 400 to 600 bp. The DNA was then enriched using the Arima-HiC v2 kit Enrichment beads. Using the NEBNext Ultra II DNA Library Prep Kit (New England Biolabs) for end repair, a-tailing, and adapter ligation. This uses a custom protocol which resembles the standard NEBNext Ultra II DNA Library Prep protocol but where library preparation occurs while DNA is bound to the Enrichment beads. For library amplification, 10 to 16 PCR cycles were required, determined by the sample biotinylation percentage. The Hi-C sequencing was performed using paired-end sequencing with a read length of 150 bp on an Illumina NovaSeq X instrument.

### Genome assembly, curation and evaluation

***Assembly***

Prior to assembly of the PacBio HiFi reads, a database of *k*-mer counts ( *k* = 31) was generated from the filtered reads using FastK. GenomeScope2 ( Ranallo-Benavidez *et al.*, 2020) was used to analyse the *k*-mer frequency distributions, providing estimates of genome size, heterozygosity, and repeat content.

The HiFi reads were first assembled using Hifiasm ( Cheng *et al.*, 2021) with the --primary option. Haplotypic duplications were identified and removed using purge_dups ( Guan *et al.*, 2020). The Hi-C reads were mapped to the primary contigs using bwa-mem2 ( Vasimuddin *et al.*, 2019). The contigs were further scaffolded using the provided Hi-C data ( Rao *et al.*, 2014) in YaHS ( Zhou *et al.*, 2023) using the --break option for handling potential misassemblies. The scaffolded assemblies were evaluated using Gfastats ( Formenti *et al.*, 2022), BUSCO ( Manni *et al.*, 2021) and MERQURY.FK ( Rhie *et al.*, 2020).

The mitochondrial genome was assembled using MitoHiFi ( Uliano-Silva *et al.*, 2023), which runs MitoFinder ( Allio *et al.*, 2020) and uses these annotations to select the final mitochondrial contig and to ensure the general quality of the sequence.

***Assembly curation***

The assembly was decontaminated using the Assembly Screen for Cobionts and Contaminants (ASCC) pipeline (article in preparation). Flat files and maps used in curation were generated in TreeVal ( Pointon *et al.*, 2023). Manual curation was primarily conducted using PretextView ( Harry, 2022), with additional insights provided by JBrowse2 ( Diesh *et al.*, 2023) and HiGlass ( Kerpedjiev *et al.*, 2018). Scaffolds were visually inspected and corrected as described by Howe *et al.* (2021). Any identified contamination, missed joins, and mis-joins were corrected, and duplicate sequences were tagged and removed. Sex chromosomes were identified by synteny analysis. The curation process is documented at https://gitlab.com/wtsi-grit/rapid-curation (article in preparation).

***Assembly quality assessment***

The Merqury.FK tool ( Rhie *et al.*, 2020), run in a Singularity container ( Kurtzer *et al.*, 2017), was used to evaluate *k*-mer completeness and assembly quality for the primary and alternate haplotypes using the *k*-mer databases ( *k* = 31) that were computed prior to genome assembly. The analysis outputs included assembly QV scores and completeness statistics.

A Hi-C contact map was produced for the final version of the assembly. The Hi-C reads were aligned using bwa-mem2 ( Vasimuddin *et al.*, 2019) and the alignment files were combined using SAMtools ( Danecek *et al.*, 2021). The Hi-C alignments were converted into a contact map using BEDTools ( Quinlan & Hall, 2010) and the Cooler tool suite ( Abdennur & Mirny, 2020). The contact map is visualised in HiGlass ( Kerpedjiev *et al.*, 2018).

The blobtoolkit pipeline is a Nextflow port of the previous Snakemake Blobtoolkit pipeline ( Challis *et al.*, 2020). It aligns the PacBio reads in SAMtools and minimap2 ( Li, 2018) and generates coverage tracks for regions of fixed size. In parallel, it queries the GoaT database ( Challis *et al.*, 2023) to identify all matching BUSCO lineages to run BUSCO ( Manni *et al.*, 2021). For the three domain-level BUSCO lineages, the pipeline aligns the BUSCO genes to the UniProt Reference Proteomes database ( Bateman *et al.*, 2023) with DIAMOND blastp ( Buchfink *et al.*, 2021). The genome is also divided into chunks according to the density of the BUSCO genes from the closest taxonomic lineage, and each chunk is aligned to the UniProt Reference Proteomes database using DIAMOND blastx. Genome sequences without a hit are chunked using seqtk and aligned to the NT database with blastn ( Altschul *et al.*, 1990). The blobtools suite combines all these outputs into a blobdir for visualisation.

The blobtoolkit pipeline was developed using nf-core tooling ( Ewels *et al.*, 2020) and MultiQC ( Ewels *et al.*, 2016), relying on the Conda package manager, the Bioconda initiative ( Grüning *et al.*, 2018), the Biocontainers infrastructure ( da Veiga Leprevost *et al.*, 2017), as well as the Docker ( Merkel, 2014) and Singularity ( Kurtzer *et al.*, 2017) containerisation solutions.

Table 4 contains a list of relevant software tool versions and sources.

**Table 4.** Software tools: versions and sources.

Software tool	Version	Source
BEDTools	2.30.0	https://github.com/arq5x/bedtools2
BLAST	2.14.0	ftp://ftp.ncbi.nlm.nih.gov/blast/executables/blast+/
BlobToolKit	4.3.9	https://github.com/blobtoolkit/blobtoolkit
BUSCO	5.5.0	https://gitlab.com/ezlab/busco
bwa-mem2	2.2.1	https://github.com/bwa-mem2/bwa-mem2
Cooler	0.8.11	https://github.com/open2c/cooler
DIAMOND	2.1.8	https://github.com/bbuchfink/diamond
fasta_windows	0.2.4	https://github.com/tolkit/fasta_windows
FastK	427104ea91c78c3b8b8b49f1a7d6bbeaa869ba1c	https://github.com/thegenemyers/FASTK
Gfastats	1.3.6	https://github.com/vgl-hub/gfastats
GoaT CLI	0.2.5	https://github.com/genomehubs/goat-cli
Hifiasm	0.19.8-r603	https://github.com/chhylp123/hifiasm
HiGlass	44086069ee7d4d3f6f3f0012569789ec138f42b84a a44357826c0b6753eb28de	https://github.com/higlass/higlass
Merqury.FK	d00d98157618f4e8d1a9190026b19b471055b22e	https://github.com/thegenemyers/MERQURY.FK
Minimap2	2.24-r1122	https://github.com/lh3/minimap2
MitoHiFi	3	https://github.com/marcelauliano/MitoHiFi
MultiQC	1.14, 1.17, and 1.18	https://github.com/MultiQC/MultiQC
NCBI Datasets	15.12.0	https://github.com/ncbi/datasets
Nextflow	23.10.0	https://github.com/nextflow-io/nextflow
PretextView	0.2.5	https://github.com/sanger-tol/PretextView
samtools	1.19.2	https://github.com/samtools/samtools
sanger-tol/ascc	-	https://github.com/sanger-tol/ascc
sanger-tol/ blobtoolkit	0.5.1	https://github.com/sanger-tol/blobtoolkit
Seqtk	1.3	https://github.com/lh3/seqtk
Singularity	3.9.0	https://github.com/sylabs/singularity
TreeVal	1.2.0	https://github.com/sanger-tol/treeval
YaHS	1.2a.2	https://github.com/c-zhou/yahs

### Wellcome Sanger Institute – Legal and Governance

The materials that have contributed to this genome note have been supplied by a Darwin Tree of Life Partner. The submission of materials by a Darwin Tree of Life Partner is subject to the **‘Darwin Tree of Life Project Sampling Code of Practice’**, which can be found in full on the Darwin Tree of Life website here. By agreeing with and signing up to the Sampling Code of Practice, the Darwin Tree of Life Partner agrees they will meet the legal and ethical requirements and standards set out within this document in respect of all samples acquired for, and supplied to, the Darwin Tree of Life Project.

Further, the Wellcome Sanger Institute employs a process whereby due diligence is carried out proportionate to the nature of the materials themselves, and the circumstances under which they have been/are to be collected and provided for use. The purpose of this is to address and mitigate any potential legal and/or ethical implications of receipt and use of the materials as part of the research project, and to ensure that in doing so we align with best practice wherever possible. The overarching areas of consideration are:

•    Ethical review of provenance and sourcing of the material

•    Legality of collection, transfer and use (national and international)

Each transfer of samples is further undertaken according to a Research Collaboration Agreement or Material Transfer Agreement entered into by the Darwin Tree of Life Partner, Genome Research Limited (operating as the Wellcome Sanger Institute), and in some circumstances other Darwin Tree of Life collaborators.

## Data Availability

European Nucleotide Archive: Anaspis regimbarti. Accession number PRJEB71244; https://identifiers.org/ena.embl/PRJEB71244. The genome sequence is released openly for reuse. The *Anaspis regimbarti* genome sequencing initiative is part of the Darwin Tree of Life (DToL) project. All raw sequence data and the assembly have been deposited in INSDC databases. The genome will be annotated using available RNA-Seq data and presented through the Ensembl pipeline at the European Bioinformatics Institute. Raw data and assembly accession identifiers are reported in Table 1 and Table 2.

## Author information

Members of the Natural History Museum Genome Acquisition Lab are listed here: https://doi.org/10.5281/zenodo.12159242.

Members of the Darwin Tree of Life Barcoding collective are listed here: https://doi.org/10.5281/zenodo.12158331.

Members of the Wellcome Sanger Institute Tree of Life Management, Samples and Laboratory team are listed here: https://doi.org/10.5281/zenodo.12162482.

Members of Wellcome Sanger Institute Scientific Operations: Sequencing Operations are listed here: https://doi.org/10.5281/zenodo.12165051.

Members of the Wellcome Sanger Institute Tree of Life Core Informatics team are listed here: https://doi.org/10.5281/zenodo.12160324.

Members of the Tree of Life Core Informatics collective are listed here: https://doi.org/10.5281/zenodo.12205391.

Members of the Darwin Tree of Life Consortium are listed here: https://doi.org/10.5281/zenodo.4783558.
