# Supplementary material for: The genome sequence of the false flower beetle, Anaspis regimbarti Schilsky, 1895
Source: Wellcome Open Res. 2025 Feb 19;10:85. [Version 1] doi: 10.12688/wellcomeopenres.23737.1 (PMC11950724; doi:10.12688/wellcomeopenres.23737.1)
Supplement: Peer review history

# Reviewer report: Reviewer response for version 1

**DOI:** 10.21956/wellcomeopenres.26185.r120343

**Published:** 2025-03-27

**Authors:** Nikoletta Andrea Nagy

**Affiliations:** 1 University of Debrecen, Debrecen, Hungary

The manuscript is well written and the genome presented is of high quality and can contribute to future research on false flower beetles which is a diverse and interesting group. I would highlight that the methods and the tools used are described clearly and thoroughly.

 I have only one suggestion for the authors, specifically to include the reason for choosing this particular species for genome sequencing. As described very well, the whole family can be of interest for future directions but it is not mentioned why the presented beetle was selected for this study.

Are sufficient details of methods and materials provided to allow replication by others?

Yes

Is the rationale for creating the dataset(s) clearly described?

Partly

Are the datasets clearly presented in a useable and accessible format?

Yes

Are the protocols appropriate and is the work technically sound?

Yes

Reviewer Expertise:

insect genomics, insect transcriptomics, insect behavioural genetics

I confirm that I have read this submission and believe that I have an appropriate level of expertise to confirm that it is of an acceptable scientific standard.

# Reviewer report: Reviewer response for version 1

**DOI:** 10.21956/wellcomeopenres.26185.r120348

**Published:** 2025-03-13

**Authors:** Stephan Koblmüller

**Affiliations:** 1 University of Graz, Graz, Austria

In this manuscript, Barclay et al. present the whole genome assembly of the false flower beetle, *Anaspis regimbarti*, a tenebrioid beetle from Western Palearctic. I particularly appreciate the extensive background info about the target species, something that’s rarely seen in this type of papers. However, there’s no clear rationale for sequencing this particular species; maybe add 1-2 sentences at the end of the introduction.  

 The data are clear; the methods are well described. I have no objections to any of the data presented or the results. As far as I can tell, the datasets are deposited in publicly accessible repositories.

Are sufficient details of methods and materials provided to allow replication by others?

Yes

Is the rationale for creating the dataset(s) clearly described?

No

Are the datasets clearly presented in a useable and accessible format?

Yes

Are the protocols appropriate and is the work technically sound?

Yes

Reviewer Expertise:

phylogenetics, population genetics, phylogeography, evolutionary biology

I confirm that I have read this submission and believe that I have an appropriate level of expertise to confirm that it is of an acceptable scientific standard.
